# Investigating lexical categorization in reading based on joint diagnostic and training approaches for language learners

**DOI:** 10.1038/s41539-024-00237-7

**Published:** 2024-04-10

**Authors:** Benjamin Gagl, Klara Gregorová

**Affiliations:** 1 Self-learning Systems Laboratory, Department of Special Education and Rehabilitation, University of Cologne, Cologne, Germany; 2 Department of Psychology and Sports Sciences, Goethe University, Frankfurt am Main, Germany; 3 Department of Child and Adolescent Psychiatry, Psychosomatics and Psychotherapy, University Hospital & German Center of Prevention Research on Mental Health, Würzburg, Germany; 4 Department of Psychology, Julius-Maximilians-University of Würzburg, Würzburg, Germany

**Keywords:** Reading, Education, Human behaviour

## Abstract

Efficient reading is essential for societal participation, so reading proficiency is a central educational goal. Here, we use an individualized diagnostics and training framework to investigate processes in visual word recognition and evaluate its usefulness for detecting training responders. We (i) motivated a training procedure based on the Lexical Categorization Model (LCM) to introduce the framework. The LCM describes pre-lexical orthographic processing implemented in the left-ventral occipital cortex and is vital to reading. German language learners trained their lexical categorization abilities while we monitored reading speed change. In three studies, most language learners increased their reading skills. Next, we (ii) estimated, for each word, the LCM-based features and assessed each reader’s lexical categorization capabilities. Finally, we (iii) explored machine learning procedures to find the optimal feature selection and regression model to predict the benefit of the lexical categorization training for each individual. The best-performing pipeline increased reading speed from 23% in the unselected group to 43% in the machine-selected group. This selection process strongly depended on parameters associated with the LCM. Thus, training in lexical categorization can increase reading skills, and accurate computational descriptions of brain functions that allow the motivation of a training procedure combined with machine learning can be powerful for individualized reading training procedures.

## Introduction

Reading opens a portal to nearly unlimited sources of new knowledge for an informed life. However, when one cannot read or is a slow reader, which is frequent in people with dyslexia or migrant language learners, access to written information is reduced. Such issues may lead to hampered language processing^1^ and suboptimal everyday decisions, reducing socioeconomic status (e.g., ref. ^2^). Thus, it is crucial to develop support programs to increase reading skills in slow readers. Here, we outline and evaluate such a program by studying non-native German speakers willing to learn German, a group of readers typically well below the reading rates of native speakers (e.g., refs. ^3,4^). In this study, we motivate a training procedure from a neuro-cognitive computational model, the lexical categorization model (LCM,^5^). The LCM is capable of describing the activation in the left-ventral occipital cortex that also holds the so-called visual word form area^6,7^ better than multiple alternative cognitive and neurocognitive models based on the assumption of the implementation of a lexical categorization process (see ref. ^5^ for details). Moreover, in combination, we develop individualized machine learning diagnostics bearing the potential to create an effective and individualized training procedure for slow readers. Nonetheless, based on feature importance metrics, the machine learning results allow the investigation of which processes are essential to predict training success accurately.

Fast visual word recognition is central to efficient reading. It describes transforming visual information into meaning (e.g., refs. ^8,9^). The more efficiently a reader implements visual word recognition, the better the general reading performance. This association was shown (i) in typical reading adults^10,11^, (ii) in dyslexic readers^12–14^, (iii) in language learners (L2; refs. ^15,16^), and (iv) in beginning readers, after extensive training of letter-to-sound associations^17–19^. Besides, in less proficient readers, visual word recognition becomes more critical for text comprehension^20,21^. The present study focuses on a training procedure for better word recognition to increase reading speed (e.g., refs. ^5,22^).

The development of remediation programs typically starts by motivating a training procedure from a well-evaluated model. For example, the phonics approach (e.g., see ref. ^23^ for a recent review) was developed to help young dyslexic readers by training the relationship of graphemes to their phonemes, i.e., a central process implemented in Dual-Route models^8,24^. A meta-analysis^25^ found that the program yielded a small to medium effect size, indicating that the research transfer, i.e., from a visual word recognition model to a training program, was successful. Here, we implement a cascade from model to training for the neuro-cognitive Lexical Categorization Model (LCM) to gather causal evidence for the model (e.g., ref. ^5^), motivated by the successful development of the Phonics training.

Brain imaging investigations try to establish the link between cognitive processes described in classical models and brain functions (e.g., see ref. ^26^) or motivate multiple new theories of how one implements reading in the brain (e.g., refs. ^7,26–29^). Numerous studies could reliably show an activation of the so-called visual word form area, a region in the left occipitotemporal cortex (lvOT, e.g., ref. ^6^), in response to visually presented words. Furthermore, visual word form area activity is reduced in slow readers (e.g., ref. ^30^) or illiterates (e.g., ref. ^31,32^) when seeing words and electrical stimulation in this part of the brain prevents patiens from reading (e.g., see ref. ^33^). Thus, these findings strongly indicate that the visual word form area is essential for efficient word recognition and reading.

**Fig. 1:**
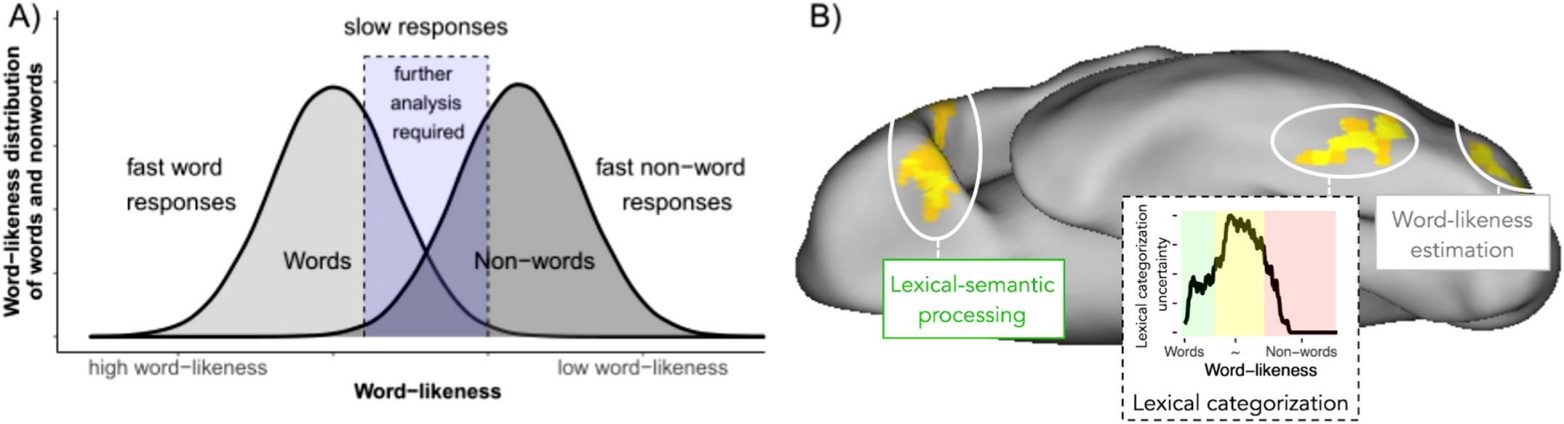
The lexical categorization process and its implementation in the left-ventral occipito temporal cortex. **A** A two-stage model for lexical decision tasks from ref. ^36^ (Figure adaptation based on Fig. 1 from the original publication). One word-likeness distribution represents words (light gray), and one distribution is for non-words (dark gray). Critical here is that behavioral responses are typically fast and accurate when the two distributions do not overlap, i.e., for highly word-like words and highly word-unlike non-words. Only when the two distributions overlap word recognition becomes slow and erroneous. **B** Schematic visualization of lexical categorization processing in the left-ventral occipito-temporal cortex assumed in the lexical categorization model from ref. ^5^ in the context of the left hemisphere of our brain, including pre- (word-likeness estimation) and post-processes (lexical-semantic processing). According to the lexical categorization model, the word-likeness of a letter string is estimated in posterior brain regions of the left ventral occipitotemporal cortex. In a more anterior region, near the so-called visual word form area, the word-likeness estimation is used to categorize the letter string as meaningful or not to prevent effortful processes of meaning extraction from non-words in more anterior regions. When inspecting the black curve in the figure, one can see that the lexical categorization uncertainty has a non-linear inverted U-shape relationship with word likeness. Compared to the model presented in **A**, we see that the uncertainty is low for word-like words and highly word-unlike non-words (Green and red areas, respectively). Only, at intermediate word-likeness estimates, uncertainty is high and categorization more difficult (yellow area). Thus, readers strongly activate the visual word form area and show slow and erroneous word recognition behavior at intermediate word-likeness levels.

Theoretical proposals on which process is implemented in the visual word form area were highly valuable in bringing forward neuro-cognitive ideas of how we implement reading on a neuronal level (i.e., refs. ^7,26–29^). All were verbally descriptive without explicit implementations (for exceptions, see refs. ^5,34,35^). In a recent study^5^, we, therefore, ran an explicit comparison of the computational implementations of the cognitive Dual-Route Model (i.e., ref. ^8^) and the ad-hoc computational implementations of the existing neuro-cognitive models (i.e., refs. ^7,26–29^). In addition, we implemented a novel model that was motivated by behavioral findings (ref. ^36^; Fig. 1A) and more general principles of ventral stream organization (e.g., ref. ^37^), the Lexical Categorization Model (LCM).

Extensive model comparisons identified the LCM as an adequate model of the left-ventral occipito temporal cortex activation (Fig. 1B; ref. ^5^). The primary assumption of the LCM is that the left-ventral occipito temporal cortex implements a lexical categorization process. This process is assumed to filter non-words from further consideration for lexical or semantic processing (i.e., distinguishing pre-lexical and lexical processing along the ventral visual stream; see refs. ^38–40^). The current model version assumes that the categorization process identifies whether a letter string is meaningful based on the word likeness of the letter string. Therefore, lexical categorization is easy for word-like words (i.e., highly familiar words) and word-un-like non-words (i.e., consonant strings) but difficult when word-likeness distributions of words and non-words overlap (Fig. 1A). Note that this pattern is not only reflected in the activation of the left-ventral occipito temporal cortex but also in behavioral response patterns (ref. ^36^; Fig. 1A) and that the word likeness estimation is based on a lexicon assumption that includes all words of German (i.e., from the SUBTLEX database; ref. ^41^). In other words, the lexical categorization uncertainty is low for high word-like words (left part of Fig. 1A and green area in the lexical categorization uncertainty curve in Fig. 1B) and low word-like non-words (right part in Fig. 1A and red area in Fig. 1B), but in between, when word and nonword distributions overlap, the lexical categorization uncertainty is high (purple area in Fig. 1A and yellow area in Fig. 1B). So, when lexical categorization is demanding and cannot be implemented only on word-likeness, we assume that additional resources (e.g., spelling information) aid the process of solving the lexical categorization (i.e., differentiate between meaningless or meaningful letter strings).

Critical for interpreting these results is that the LCM has no free parameter. Hence, all simulations are highly transparent, which is typically not the case for other model-based analyses of lvOT activation (e.g., refs. ^42,43^). The critical advantages of a transparent model for remediation research is twofold: (i) One can directly motivate a training program from the central cognitive processes represented by the model, and (ii) one can build a prediction model (i.e., for individualized diagnostics) that achieves high transparency by including meaningful features (e.g., that reflect processes implemented in a well-evaluated model) and the application of explainable machine learning methods (i.e., feature importance metric; e.g., ref. ^44^). These advantages are beneficial as one can evaluate whether the intervention targets the process assumed to be trained. From a more general neuro-cognitive perspective, this deducing technique of transparent predictions is a way to investigate the causal relevance of proposed brain functions for behavior^45^.

**Fig. 2:**
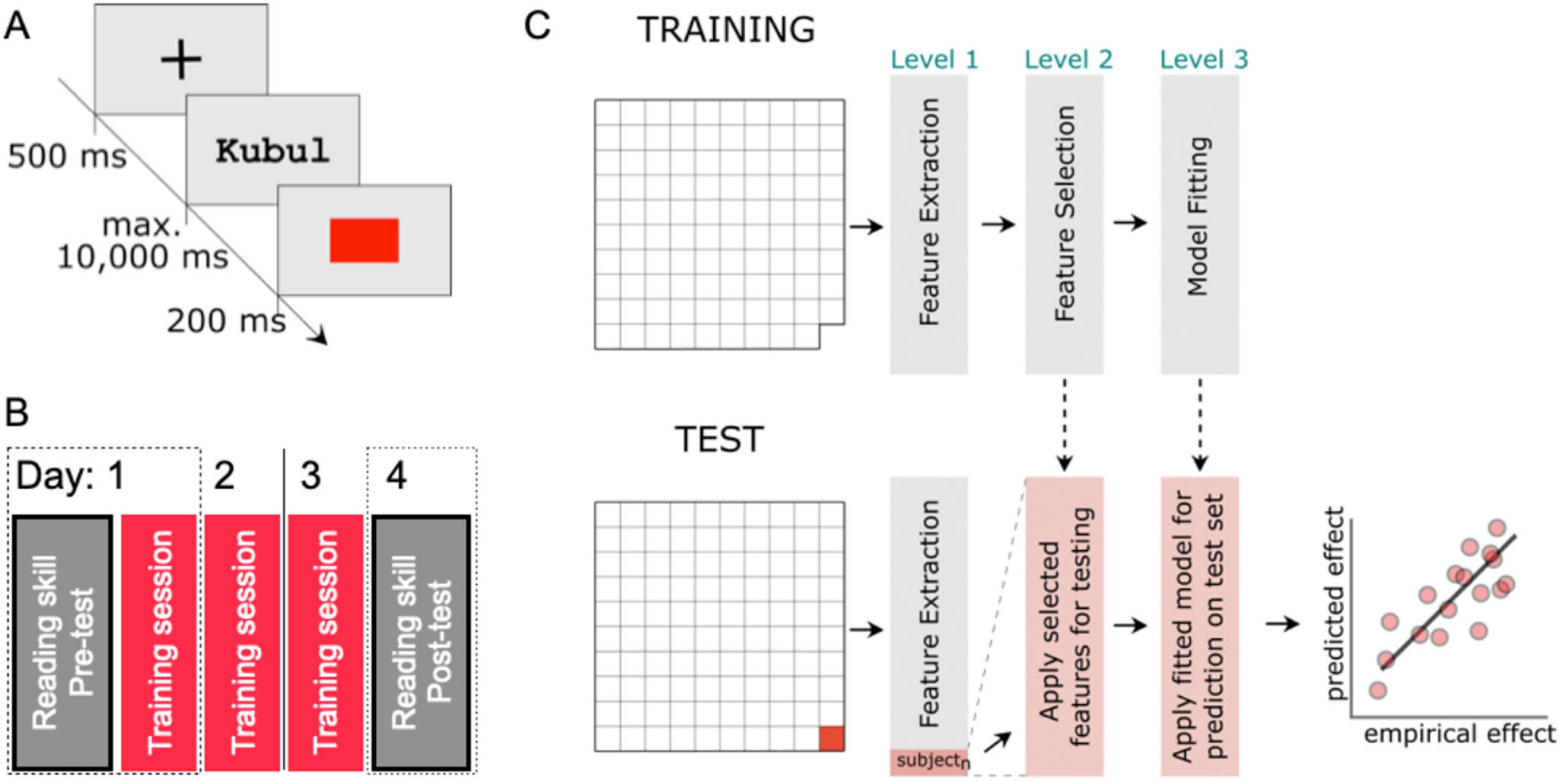
Training procedure and schematic description of the machine learning pipeline for the individualized diagnostics. **A** Example of one trial from the lexical categorization training. First, a fixation cross is presented, followed by a letter string that is presented until a button press, but for a maximum of 10 s. After a response, feedback is given. A red square indicates an incorrect response (either a word was categorized as a nonword or vice versa), and a green square indicates a correct response. Participants pressed the “f” key for a word categorization and the “j” key for a nonword categorization. **B** The training design comprises three sessions and a pre-post diagnostic based on a standardized reading speed test (adult version of the Salzburger Lesescreening - SLS; ref. ^71^). The pre-test and the first session of the lexical categorization training (dashed box) were used to predict the outcome (dotted box). In Experiments 2 and 3, the same procedure was implemented for control training procedures (Phonics and an adapted lexical categorization training). In Experiment 1, the post-test was conducted after the last training session on day 3. **C** Analysis steps in the applied cross-validation for the training procedure (upper row) and testing procedure (lower row). The grid on the left represents the datasets. We used a consensus nested leave-one-out-cross-validation; thus, for training, we used all but one dataset to generate features, select features, and train a prediction model. We then applied the trained pipeline to the one left-out dataset for testing. Note we used this cross-validation procedure to prevent extensive overfitting (see methods and supplement for more details).

Thus, we aim to predict the outcome of a 3-day lexical categorization training procedure (Fig. 2A, B; i.e., based on a lexical decision task with feedback). The training aims to increase lexical categorization capabilities, i.e., the process likely implemented in the left-ventral occipito temporal cortex. Our participants were German learners, of which most showed a training effect on their reading speed (i.e., Experiment 3 in ref. ^5^). To accurately predict the training outcome, we implemented a machine learning pipeline based on the parameters of the LCM (e.g., lexical categorization uncertainty, word-likeness operationalized here as the Orthographic Levenshtein Distance 20; OLD20^46^; see Fig. 1B) and other potentially relevant features (e.g., incoming reading speed) to define an optimal prediction model. These features reflect if potential word recognition processes are operating for each reader. We measured this based on data from the initial training session and parameters associated with the cognitive processes by random effect estimates from linear mixed models^47^. For example, we estimated each letter string’s lexical categorization uncertainty based on the lexical categorization model^5^. After that, we included the parameter in a linear mixed model that predicted the lexical decision response times in the initial training session. Crucially, we included lexical categorization uncertainty not only as a fixed effect in the linear mixed model but also as a random slope estimate on the random effect of participants. The latter allowed us to extract the interindividual variability of the effect of lexical categorization uncertainty and other parameters (e.g., OLD20, Lexicaliy). Estimating the lexical categorization uncertainty effect as a random slope for each participant allows the estimation of effect sizes for each participant in the context of a larger regression model. Note that one can implement this only in a linear mixed model including crossed-random effects (in our case, we include participant and stimulus as random effects simultaneously). The resulting features are the basis for the individualized diagnostic based on machine learning and, therefore, can be investigated based on feature importance metrics (e.g., ref. ^44^).

We added two alternative training approaches to contextualize the Lexical Categorization training effects on reading performance. Experiments 2 and 3 implemented randomized controlled trials based on tasks that included the same stimuli as in the lexical categorization training (i.e., same letter strings). Experiment 2 implemented phonics training based on a letter-based phonological awareness task that was effective in helping dyslexic readers^25,48^. Here, we trained the grapheme-phoneme associations of language learners. The central task of participants is to indicate if a grapheme associated with a presented phoneme (i.e., letter sound) is included in a letter string (i.e., word or nonword) presented simultaneously (e.g., is /s/ is present in house). We are aware that our group is different from the typical target group, as the readers have been fluent in their first language. Nonetheless, we have yet to be aware of a study investigating whether Phonics training helps language learners. Still, the phonics training might increase reading performance if readers are unfamiliar with transparent grapheme-phoneme associations of German.

In Experiment 3, we implement a variant of the Lexical Categorization training that simultaneously trains the lexical categorization mechanism and the formation of the underlying representation. We recently found that readers implement prediction error representation (e.g., refs. ^27,49,50^) on the visual level with orthographic properties (i.e., the orthographic prediction error; refs. ^34,51^). The orthographic prediction error representation results from the computation we implemented on the level of pixels (i.e., the smallest units of an image) that integrates the probability of a pixel being informative, estimated based on all known words, with the presented word at each trial. This measure focuses on the pixel-level information that allows letter distinction (e.g., dot on the *i* or the lower part of the *g*) and, at the same time, ignores the visual information that is redundant across letters (e.g., the vertical line on the left in *M, N, B, D, F, H, K, L, P, U, R, E*). The conception of this training procedure was to combine the effects of better lexical categorization and the formation of orthographic representations. This integration of the two mechanisms is reasonable, as conceptually, the visual-orthographic representations could inform the categorization process, and training both is expected to increase further the positive effects of the training on reading performance. We trained the formation of visual-orthographic representations by repeatedly changing the visual appearance of the letter strings by changing the font (i.e., 50 blocks of 32 strings with the same font). This procedure determines an adaptation process on the visual level, as the new font could be in italics, so one needs to account for the tilt in the letters (i.e., word vs. *word*). Consequently, we expected that training this adaptation process and lexical categorization simultaneously increases the effect on reading performance compared to the Lexical Categorization training.

Thus, the present study presents the Lexical Categorization training effects contextualized with the effects from control training procedures and a machine learning-based diagnostic procedure that tries to predict the Lexical Categorization training outcome. For the application of the machine learning diagnostics procedure, we expect that the individual pre-training performance in lexical categorization determines the success of the lexical categorization training. In addition, we investigated the importance of the individual features based on (i) the number of selections in all evaluated pipelines and (ii) *t* values when investigating the best-performing solution. Here, we expect the features related to the LCM to be essential.

## Results

### Training results

In ref. ^5^, we initially described the lexical categorization training effect and found a significant improvement in the reading speed by 23% after three training sessions. We also showed that the lexical categorization training specifically reduced the lexical categorization difficulty effect. To measure this reduction, we used lexical decision times from the training sessions and estimated the interaction of the lexical categorization difficulty with the training session. The significant association between the reading speed increase and the individually estimated interaction of lexical categorization difficulty and training session showed a positive correlation. The correlation indicates that the lexical categorization training directly trained the lexical categorization process, leading to increased reading speed. In the following, we will first provide a more detailed investigation of the training effects before we provide the results from the machine learning-based diagnostic approach.

**Fig. 3:**
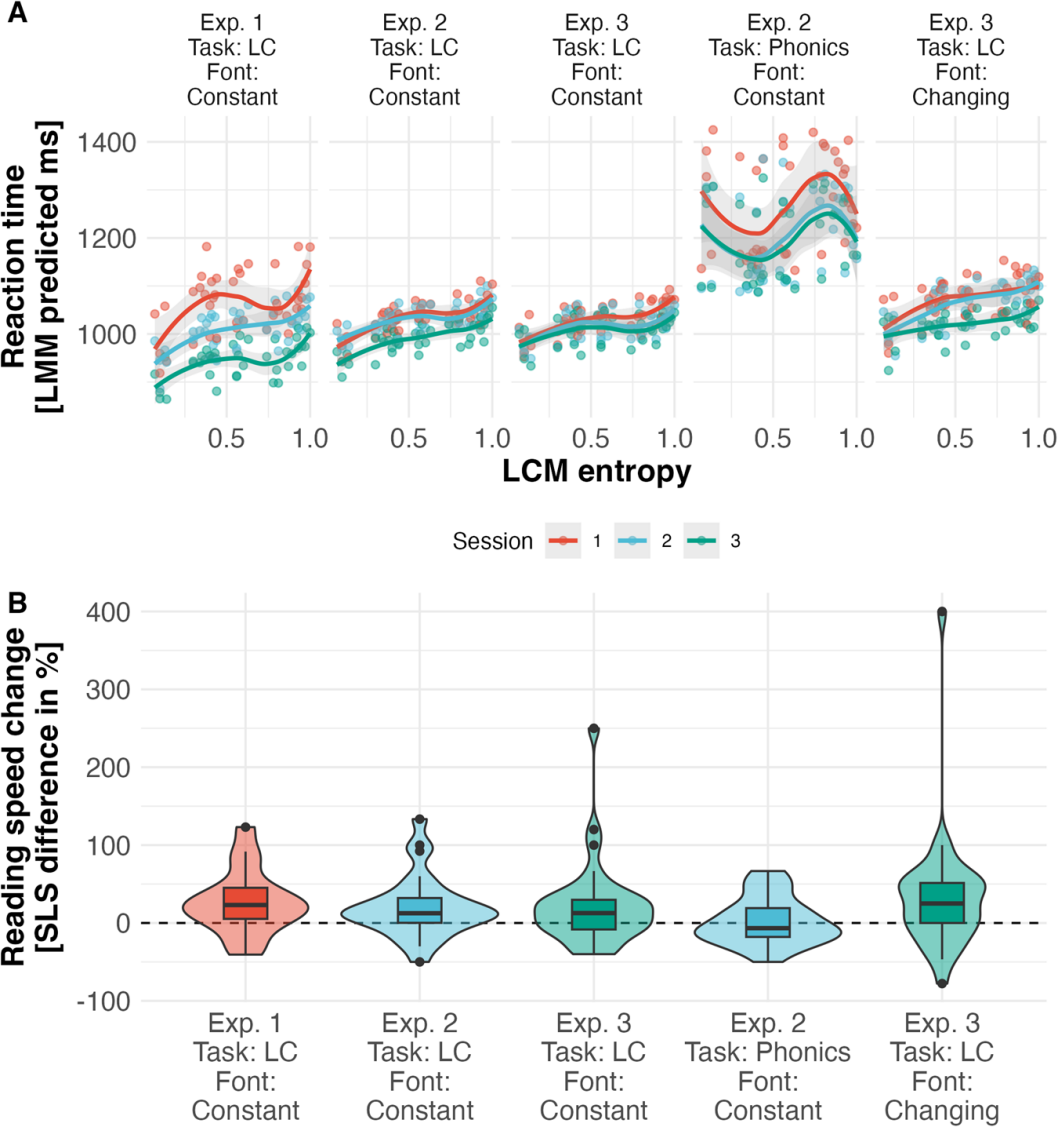
Results from behavioral training study. **A** Reaction times for three sessions of all three experiments, including the phonics and font-change control tasks, relative to the lexical categorization model entropy parameter. We present the reaction times corrected for the effects of other word characteristics (Word frequency, lexicality, effect of errors, and stimulus order effects) based on predictions from the fitted linear mixed regression models used for statistical analysis. The model-based reaction times are aggregated across the session and entropy by mean. The light gray area reflects the 95% confidence interval. **B** Reading speed change in percent relative to pre-training reading speed measured by SLS reading speed test (adult version of the Salzburger Lesescreening; ref. ^71^). For all boxplots, the horizontal lines represent the median, the boxes represent data from the 25th to the 75th percentile, and the whiskers extend up to 1.5 times the interquartile range. Dots show participants with extream values and violin plots show the distribution of the training effects on the level of participants.

In contrast to the previous analysis, we here focus on the presentation of the single experiments to show that the training effects are not specific to a study but replicated across studies. In Fig. 3, we separately present the effect of training on reading speed for the three training studies. In addition, Table 1 shows that lexical categorization training effects are significant, indicating high replicability of the effect. In detail, the response times analysis of the training sessions consistently showed an increase with categorization uncertainty (see Fig. 3B; i.e., response times are highest when the categorization uncertainty is largest). Thus, in all three experiments, we found a significant effect of the LCM estimated lexical categorization uncertainty (Fixed effect: FE_*Exp*.1/2/3_ = 0.16/0.12/0.12; Standard error: SE_Exp.1/2/3_ = 0.01/0.01/0.01; *t* value: *t*_*Exp*.1/2/3_ = 11.32/10.02/9.78) and training session (FE_Exp.1/2/3_ = −0.07/−0.04/-0.03; SE_Exp.1/2/3_ = 0.00/0.00/0.00; *t*_Exp.1/2/3_ = 17.61/12.60/7.871). Note that only in Experiment 1 we could identify a signification reduction of the lexical categorization uncertainty effect with a training session (FE_Exp.1/2/3_ = −0.21/−0.00/−0.01; SE_Exp.1/2/3_ = 0.02/0.01/0.01; *t*_Exp.1/2/3_ = 2.59/0.09/1.581).

**Table 1:** Pre-to-post changes in the overall reading speed measured by the SLS in percent as shown in Fig. 3B, including statistical comparison against zero (Significant effect sizes presented in bold numerals)

Experiment	Condition	M	SD	*t*	DF	*p*
1	Lexical categorization (LC) training	**26.6** (**20.5**)	41.8 (34.6)	2.62 (2.37)	16 (15)	.018 (.035)
2	LC training	**20.4** (**16.1**)	40.2 (33.9)	2.65 (2.42)	26 (25)	.014 (.023)
2	Phonics training	2.5 (0.5)	30.4 (28.1)	0.43 (0.01)	26 (25)	.670 (.992)
3	LC training	**22.4** (**15.0**)	54.9 (36.4)	2.31 (2.30)	31 (30)	.028 (.029)
3	LC font training	**33.2** (**21.4**)	79.0 (43.6)	2.38 (2.79)	31 (30)	.024 (.009)

Also, in paratheses, we show the same results but with an outlier correction that removes all participants ± 2 standard deviations as defined in the preregistration of Study 3. Note all *t*-test comparisons have been two-sided, and the effect size was estimated based on percent change pre-post training.

Experiments 2 and 3 included two alternative training procedures (i.e., randomized controlled trials). In Experiment 2, we compared the lexical categorization training against classical phonics training based on a letter-based phonological awareness task^25,48^. The phonics training did not significantly increase reading speed (see Fig. 3B and Table 1). Comparing lexical categorization and phonics in Experiment 2, we found no significant difference, as the phonics had a small but unreliable positive effect (*t*(26) = 1.73, *p* = 0.096). Also, when comparing the lexical categorization and phonics training reaction time, we found longer durations and no lexical categorization effect in the phonics training (cp. Fig. 3A; Lexical Categorization uncertainty effect: FE = −0.00, SE = 0.01, *t* = −0.40). Nonetheless, the reaction times increased with decreasing word-likeness (FE = 0.02, SE = 0.00, *t* = 6.01), indicating easier phoneme detection for words. Also, we found a significant reduction in response times with training sessions (FE = −0.09, SE = 0.00, *t* = −31.92).

In Experiment 3, the control training procedure was a variant of the lexical categorization training: the lexical categorization font training. Here, again, participants had to implement a lexical decision task with identical word and nonword stimuli, but in this training, the font changed every 32 trials. This adaptation was motivated by the finding of an orthographic prediction error^34^, indicating that readers adapt to the visual appearance of words to form efficient orthographic representations while reading. The lexical categorization font training also resulted in a significant reading speed increase (see Table 1). However, the effect was not higher than for the lexical categorization training (*t*(31) = −0.61, *p* = 0.54, see Table 1). Like in the lexical categorization training, the response times showed a significant lexical categorization uncertainty effect (FE = 0.12, SE = 0.01, *t* = 9.74), session effect (FE = −0.03, SE = 0.01, *t* = 5.60), and interaction effect of lexical categorization uncertainty and session (FE = 0.02, SE = 0.01, *t* = 3.97, see Fig. 3A). In line with hypotheses and analysis plans from the preregistration, in lexical categorization font training, the lexical categorization effect was trainable, while this improvement was modulated by word-likeness (see Supplementary Table 2; for results of lexical categorization training from all lexical categorization training data combined). Note that the design difference likely determines the higher training effect of the first study as we find a significant effect of training week showing a lower training effect in the second week (Mean difference: 31%; *t*(31) = −2.93, *p* = 0.00452). This difference was not modulated by the reading speed of the pre-assessment or the reaction times of the first training session (all *ts* < 1.3).

In three separate experiments, these findings show that the lexical categorization process is trainable. Also, the lexical categorization training has a beneficial effect on the overall reading speed of readers learning German. Still, training benefits show substantial interindividual variability, with about 30.26% of the current sample showing no improvement (Training effect ≤0). For an efficient implementation of the lexical categorization training, identifying non-responders in advance would allow focusing resources on responders. Thus, implementing a diagnostic procedure before training could benefit the training outcomes as resources can focus on the slow-reading individuals who likely respond.

### Prediction of lexical categorization training effects

**Fig. 4:**
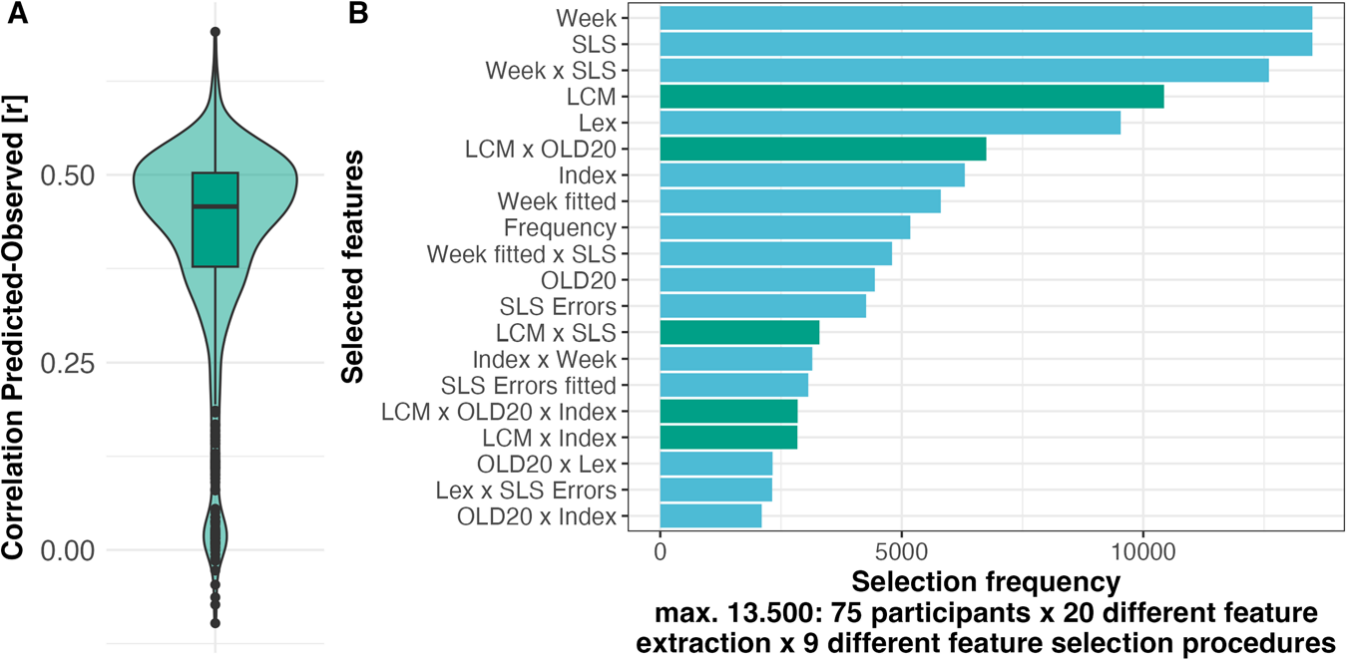
Overall analysis of the correlation between predicted and observed training effects, including an analysis of feature importance. **A** Distribution of predicted vs. observed correlations resulting from leave-one-out-cross-validation across all variations of the machine learning pipeline. **B** The most frequently selected features among the 20 feature extraction models and nine different feature selection procedures. As features are selected 75 times for each participant due to the leave-one-out-cross-validation, the maximal frequency is 13,500. Only the top 20 features with the highest occurrences are displayed here. The green color highlights the LCM-related features.

We will use the correlation between predicted vs. observed training effect (i.e., based on reading speed increase) to evaluate the variation in the implemented machine learning pipelines. We established the prediction model based on leave-one-out-cross-validation (see methods for detailed description). The correlations from all tested machine learning pipelines ranged from −0.10 to 0.69, with a mean of 0.42 and a mode just below 0.5 (Fig. 4A). When we aggregate the predicted values from all pipelines for each participant (median across 720 predictions), the correlation between predicted vs. observed training effect was moderate (*r* = 0.58; *t*(73) = 6.02, confidence interval: 0.40–0.71, *p* < 001). The variation of the correlation (Fig. 4A) indicates that the quality of the prediction varied based on the combination of feature extraction procedures (model structures and predictors of hierarchical regressions), feature selection procedures (extent of interindividual consensus on the relevance of features), and prediction models (linear regressions, support vector machine, random forest) but overall, independent of the procedure, we can predict the reading speed with good accuracy (see Supplement for differential analyses). When inspecting the feature importance (Fig. 4B), based on how often the stepwise regression selected a feature (for details, see Methods), we find that test week (Week; i.e., from the randomization) and incoming reading speed (SLS), including the interaction, were essential predictors that have been selected for all pipelines. Note that the more often a feature is selected indicates that they are vital for accurately predicting the lexical categorization training effect. After these more general measures, the most selected word characteristic-based feature was the lexical categorization uncertainty. We expected that the predictive power of the lexical categorization uncertainty would be high as participants trained lexical categorizations and that response times data showed interactions of lexical categorization uncertainty with training (see ref. ^5^ or above; all LCM-related features are highlighted in green in Fig. 4B). Thus, this finding indicates the importance of the lexical categorization uncertainty effect in the initial session for predicting later training success.

### Best prediction model

**Fig. 5:**
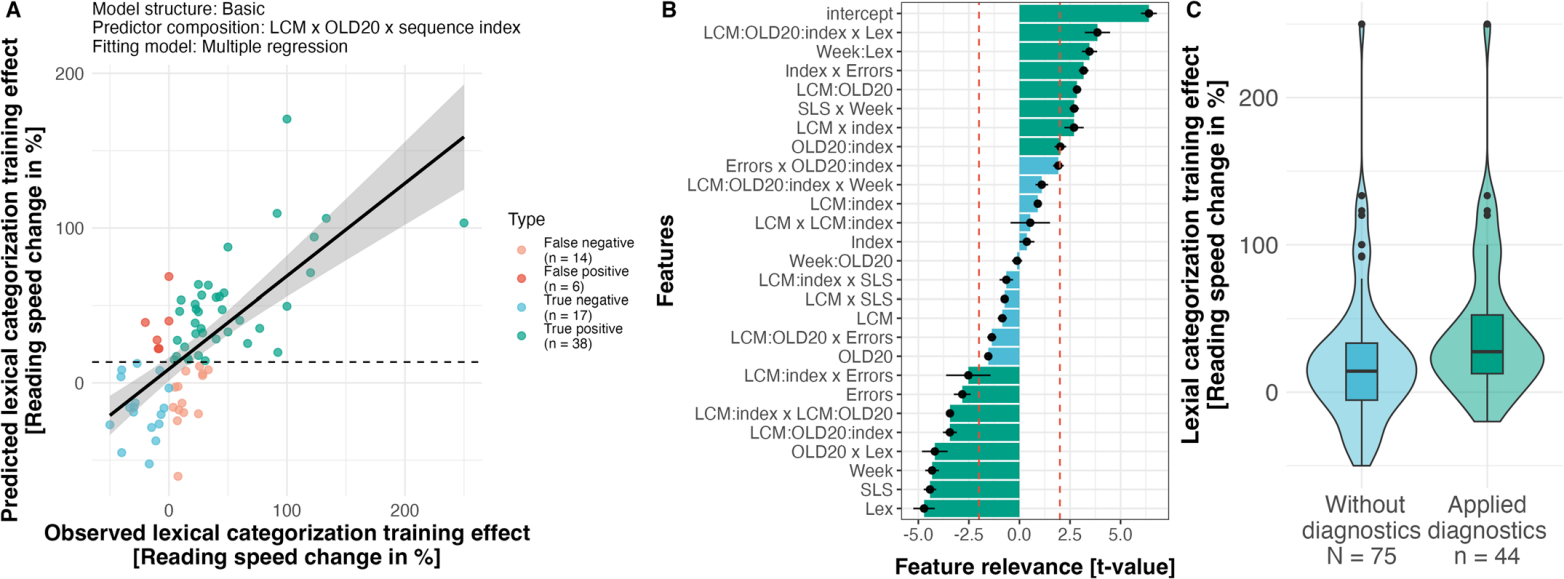
Inspection of the best prediction model. **A** Correlation of the predicted and observed reading speed difference, pre/post training from the best prediction model. The dashed line indicates the decision boundary (i.e., cases with >13.5% predicted reading speed increase are considered responders, established based on a sensitivity of 0.73 and specificity of 0.74; see Supplementary Fig. 2), and colors mark the true positive (Correctly predicted responders), true negative (Correctly predicted non-responders), false positive (Incorrectly predicted non-responders), and false negative cases (Incorrectly predicted responders). The black line indicates the overall correlation, and the gray area reflects the 95% confidence interval. **B** Feature relevance of all included features based on *t* values (i.e., signed median and standard deviations across 75 cross-validation runs including significance markers, red dashed line > 2 or < −2). LCM: lexical categorization model uncertainty effect, Lex: lexicality effect, Index: sequence index effect, OLD20: word-likeness effect based on the OLD20 measures^46^, Frequency: word frequency effect based on SUBTLEX-DE measure^41^, Week: week of training effect (1 vs. 2), SLS: incoming reading speed effect (i.e., adult version of Salzburger Lesescreening^71^), Errors: Correct vs. incorrect lexical decisions in the training task, interaction sign “x'': interaction added at Level 2 during the feature selection, interaction sign “:'': interaction fitted with the random effect structure. **C** Applying the best-performing diagnostic model for categorization. Benefits in reading speed when no diagnostics applied vs. when diagnostics applied (i.e., if the training included only readers of which the model would predict learning success).

The most often selected pipeline (based on consensus nested leave-one-out cross-validation) included the multiple regression fitting procedure and a feature selection criterion of 10, combined with the predictor composition that estimated the change of the lexical categorization uncertainty and OLD20 effects within the first session (i.e., three-way interaction of lexical categorization uncertainty, OLD20, and sequence index). This pipeline results in the best prediction results in 21 out of 75 cross-validation loops. Especially, the predictor composition was consistently the best variant, resulting in the most accurate predictions in 55 out of 75 cross-validation loops. When applying these hyperparameters (i.e., multiple regression, the cutoff value of 10, and predictor composition including 3-way interaction of lexical categorization uncertainty x OLD20 x training across the session; complete formula: *log*. response times ~ *lexical categorization uncertainty* * *OLD*20 * *log*.  *sequence index* + *word frequency* + *lexicality* + *errors* + *SLS* + *training week* + (*effect* ∣ *participant*)) the final correlation of predicted vs. observed lexical categorization training effects resulted in 0.69 (*t*(73) = 8.16, confidence interval = [0.55–0.79], *p* < 0.001, see Fig. 5A), explaining nearly 50% of the variance of the training effect (*R*^2^ = 0.476).

To investigate the feature relevance, we evaluated which features were essential for predicting future reading speed improvement. The pipeline selected and fitted 25 to 26 features for each reader. Note that the feature set could differ for every participant (i.e., leave-one-out-cross-validation). To visualize feature relevance, we aggregated the *t* values of the contributing features included in the pipeline that resulted in the highest predicted vs. observed correlation. This inspection is based on the median *t* value across all 75 runs (see Fig. 5B). The stepwise regression feature extraction procedure selected the individualized estimated lexical categorization uncertainty, lexicality (Word or nonword), and word-likeness effects (i.e., measured by the Levenshtein distance; OLD20^46^);. Interestingly, these features are also highly related to the theoretical concept of the lexical categorization computation, as the word likeness is the basis for the lexical categorization that results in a word-nonword decision (i.e., lexicality; see ref. ^5^ for details).

Further essential features have been the overall learning within the first session, reflecting the reduced reaction times with trials, the number of errors, and the interactions with lexical categorization uncertainty and word likeness. Note here that we can have an interaction effect that combines one or multiple interaction features (i.e., extracted interaction effect from the first level) with other features (e.g., in Fig. 5B, LCM:Index *x* LCM:OLD20, indicates the interaction effect marked by the “*x*” of two interaction features, estimated on the first level based on linear mixed models, that marked by the “:”). In addition, training week and incoming reading level also moderated the prediction of training benefits.

### Categorization diagnostics

Here, we investigated the application of the regression model to categorize individual readers as responders or non-responders. The leave-one-out-cross-validation is optimal here as it simulates implementing an individualized diagnostic procedure. An individuum is categorized based on the above-described prediction model. The boundary for the training/no-training decision is optimized on the trade-off of sensitivity and specificity to reduce miscategorization to a minimum. Here, we identified an optimal criterion value at 13.5% or above. At this criterion, we found a sensitivity of 0.73, specificity of 0.74, accuracy of 0.73, and precision of 0.86 (see Supplementary Fig. 6; *N* = 44 out of 75 selected, true positive: 38, false positive: 6, true negative: 17, false negative: 14; see Fig. 5A color coding). Thus, 73% of the selected readers did benefit from the lexical categorization training, and 74% of the not selected readers did not benefit from the training (i.e., reading speed changes are smaller or equal to 0). Therefore, the selection of responders increased the mean reading speed from 23% to 43% on the group level (FE = 0.199, SE = 0.091, *t* = 2.188, see Fig. 5C).

## Discussion

In this study, we measured the effect size of a training program motivated by a model of the activation in the left-ventral occipito-temporal cortex in visual word recognition, i.e., the lexical categorization model (LCM; ref. ^5^). In three studies (including one preregistered study), the training resulted in better reading skills for most German language learners. In addition, we found that response times during the training showed lexical categorization uncertainty effects (i.e., higher reaction times for hard-to-categorize words and non-words) and a reduction in response times with training. Also, based on an individualized diagnostic procedure using machine learning and LCM parameters, we could predict the outcome of the lexical categorization training with good precision and found that the LCM-related parameters were essential for an accurate prediction. This application may allow focusing training resources on responders only. Here, we provide and evaluate a new framework for investigating visual word recognition processes, including a possible way for practical applications. This framework uses a transparent computational model to motivate a training procedure from which training effects can be analyzed, predicted, and optimized based on explainable individualized machine learning diagnostics. Central to the diagnostic procedure is that one can use the primary model parameters from which the training originated to predict the training effects successfully.

The core assumption is that lexical categorization is a central cognitive process underlying efficient reading^5^. The goal of the lexical categorization process, as assumed by the model, is to filter out stimuli that are not meaningful and to facilitate further linguistic processing, specifically for known letter strings (see ref. ^52^ for similar conclusions). When a word is known, one extracts the meaning^8,24^ and can start to read the upcoming word and integrate words into the larger context of sentences or paragraphs^53–55^. If the letter string is unknown, a good reader might be able to infer the meaning of a word from the context, or one has to look up the meaning of that word. The latter is quite common for language learners, as integrating additional sources, like a lexicon, to learn the meaning of a word is an essential part of language learning. Thus, visual word recognition will be efficient when the categorization is fast and accurate, as the reader can initiate the consequential processes more efficiently.

Multiple LCM evaluations with native readers of German showed that LCM simulation outperforms assumptions from other models (e.g., refs. ^7,26,27,29^) that have been proposed to explain the activation patterns in the in the left-ventral occipito-temporal cortex. To test the validity of this core LCM assumption, we assumed that when reading is slow, the lexical categorization performance could be low, so improving lexical categorizations should result in better reading^5^. In an initial investigation, we showed that the training procedure resulted in a significant reading speed increase correlated with increased lexical categorization performance^5^. The findings of the present study (i.e., replicated lexical categorization training effects and high feature importance of LCM parameters, i.e., lexical categorization uncertainty, for predicting training success) are an additional indication of a direct association between lexical categorization and efficient reading.

*Lexical categorization training effects and control training*. Here, we provided a detailed description of the three lexical categorization training experiments, including the effects of alternative training procedures. The lexical categorization training increased the reading speed consistently. In contrast, the phonics control training of Experiment 2 did not increase reading skills and showed a different response time pattern. Still, we found a strong learning effect on response times. This finding suggests that the training task and not the stimuli (i.e., identical in both training procedures) are responsible for the reading speed increase of the lexical categorization training. A potential reason for the null effect of the phonics training could be our participant group. The slow readers of the present study did not have developmental deficits, only limited German language knowledge. Phonics studies successfully trained readers with developmental reading problems (e.g., Dyslexia; refs. ^23,25^). Nonetheless, there have been several readers who benefited from the training. With more data, one could implement an individualized diagnostic procedure to identify specifically the phonics responders of the group in advance.

We detected the strongest training effect for the adapted lexical categorization training procedure that included a font-change manipulation (6–11% larger effects). Also, the response time pattern showed an effect of lexical categorization uncertainty. Central here is considering a predictive mechanism on the visual level^34^ that is trained when fonts repeatedly change during the lexical categorization training, and the process has to adapt to each font. Thus, the higher increase in reading skills after training might indicate that one can also train predictive processes on the visual level. Still, this investigation needs to be replicated in the future. Overall, the lexical categorization training procedures would extend the current training procedures, providing another opportunity for slow readers to increase their reading skills.

*Individualized diagnostic procedure for the lexical categorization training*. Similar to this study, training procedures have been motivated by well-evaluated cognitive concepts (e.g., phonics; ref. ^25^). Besides, computational implementations of dual-route models have likewise been used to extract individual-level estimates for individualized diagnostics^56,57^. Note, in contrast to the machine learning approach here, the dual-route model diagnostics have the benefit that the models can simulate visual word recognition entirely, which is not the case for the neuro-cognitive approaches currently available (e.g., see ref. ^58^ for an initial step in the direction of a full neuro-cognitive visual word recognition model). We extended these efforts by drawing inspiration from a neuro-cognitive model and explicitly using model parameters to develop individualized diagnostics. The diagnostics use explainable machine learning techniques that integrate the model with training procedures and personalized assessments. Thus, the ultimate objective is to predict reading skill improvement accurately. Based on this pipeline, we predicted the change in the reading speed of each reader, pre-post training, with good accuracy. Also, when using the numeric estimates of reading speed change to categorize readers into responders and non-responders, we could increase the training effect on reading speed from 23% in the whole group (*N* = 75) to 43% (*N* = 44) in the selected group.

Using feature importance metrics, we identified that the LCM-related features were the most often selected word characteristics besides obvious candidates like incoming reading speed (the lower the incoming performance, the higher the gain through the training) and training week (lower training effects in the second week). This finding is reassuring, as one would expect that when one has difficulty implementing a lexical categorization, i.e., reflected in the behavioral performance, one should be more likely to respond to the lexical categorization training. An intriguing finding was that word frequency, a highly relevant word recognition characteristic, was selected less often^41,59^. Often, the lexical categorization uncertainty occurs in interactions with variables that are also relevant for implementing the model (e.g., with word-likeness; OLD20^46^). This finding indicates, in addition, that the underlying cognitive processes are related to the word-likeness parameters, and the lexical categorization are likely interrelated. Thus, machine learning diagnostics strongly rely on the overall LCM-based features, especially when the prediction accuracy is high.

Central to the success of the individualized diagnostics is using features based on slope estimates on the random effect of participants from linear mixed models^47^ in combination with standard features like the overall reading speed. Crucially, for the random slope estimation, we found that more complex models had no advantage in the precision of the prediction, as simpler models were preferred. One reason for this is the implementation of shrinkage in linear mixed models that removes differences in random effects estimates when models become increasingly complex^60^. Similarly, we found that more straightforward procedures, like the multiple regression method, led to the best prediction. Still, we only used a subset of methods currently available for feature selection and prediction. Several other candidate machine learning procedures may lead to better results. Gradient boosting or deep neuronal network models could be candidates in future investigations. However, using more complex machine learning methods in this context might need a more extensive dataset (ref. ^61^, a plateau at a sample size larger than 100) or decrease the interpretability of the results (e.g., see ref. ^62^).

Prospectively, multiple training procedures can be combined into one framework. For example, one could fit the machine learning pipeline to the data we gathered from the control training procedures (e.g., phonics and the lexical categorization training with font change), intending to find the optimal training procedure for each slow reader. With the further development of new transparent neuro-cognitive computational models, we have the chance to add a new, potentially effective training program that needs to be evaluated. The resulting training data can then be used to train a machine learning model to predict the training outcome. By comparing the predictions for multiple training procedures, one can find the training that most likely has the highest increase in reading skills. Furthermore, during active learning, one has several potential other training procedures that could be used when the actual training does not result in further reading skill increase. Over time, combining training procedures could lead to a fully functioning individualized training framework, providing readers with the training they benefit most.

A limitation of the present study is that it relies on cross-validation, but, in addition, one would need an evaluation based on an independent sample to fully outrule the over-fitting of our prediction models. Still, we used consensus-nested cross-validation for feature selection and hyperparameter tuning, which is generally assumed to control over-fitting sufficiently, particularly in studies with limited sample sizes^63,64^. The only difference to the consensus-nested cross-validation in its original implementation is that the authors searched for features selected in all inner loops. Still, we are searching for a hyperparameter combination selected by most inner loops. We believe this method is justifiable for considering model stability and accuracy. Besides this argumentation, we find a median correlation of 0.57 across all hyperparameters. This strengthens the assumption that we can predict the training benefit with a medium to high correlation.

To increase the performance of the diagnostic procedure, we see two major possibilities–first, the development of new parameters that accurately describe the cognitive and neuro-cognitive processes underlying reading. Here, using interpretable parameters to make sense of the feature importance metrics is essential. Adding newly developed features related to visual processing (e.g., ref. ^51^), orthographic processing (e.g., see refs. ^65,66^), phonological (e.g., see ref. ^65^), lexical (e.g., see refs. ^67,68^), or even semantic processing^69^ could be one way to increase the performance of the machine learning-based diagnostic further. Second, in the current investigation that used randomized controlled trials in two experiments, we found that the training week was a significant predictor of performance, indicating that participants might have been less motivated. Future training approaches could implement new tools or gamification elements to increase motivation (e.g., ref. ^70^). An increase in motivation could positively influence the estimation of feature weights, as higher task involvement could make estimating the underlying processes reliable. Alternatively, participants might have been more proficient in the reading speed assessment, i.e., our primary outcome measure^71^. New investigations, thus, should use alternative methods that measure reading skills (e.g., the computerized version of the SLS; ref. ^72^) or even use the highly reliable eye-tracking measure while silent sentence reading (ref. ^3,12,73,74^; but see also ref. ^75^ for a cautious note).

In sum, neuro-cognitive computational models open new possibilities for implementing potentially effective training programs. Here, we showed that lexical categorization is essential for efficient reading on the exemplar of the lexical categorization training. The training repeatedly increased the reading skills of learners of German, including a preregistered study. The model parameters (i.e., extracted from the computational implementation) have been a vital source of information for a machine learning-based diagnostic procedure able to predict training success. Thus, a good and exact understanding of the neuro-cognitive processes involved in reading that allow a computational implementation can be the optimal origin of a successful program to increase reading skills. This proof-of-concept study showed that combining response time data, psychometric measures, and computational methods can also produce valuable predictions on the level of the individual reader. The predicted benefits of the lexical categorization training, thus, allow the optimization of training resources. Therefore, as the presented diagnostic procedure is efficient at a relatively low cost, we believe that individualized and model-based diagnostic and training programs are a valuable framework that one can extend to more training procedures (e.g., phonics) and, potentially, other groups of slow readers that show activation alternations compared to typical readers in the left-ventral occipital cortex while reading (e.g., dyslexics: ref. ^30^; illiterates: refs. ^31,32^).

## Methods

### Participants

Seventy-six adult non-native German language learners participated in the three experiments (Exp. 1: 17; Exp. 2: 27; Exp. 3: 32; 17-74 years old, M = 24.41, SD = 6.89). Note that we determined the number of participants for Experiment 3 by a power analysis described in the preregistration (https://osf.io/t58ku) based on the estimated effects of Experiments 1 and 2 (Cohens d = 0.62; Power of 92%). Participants had no history of linguistic or neurological diseases and came from 28 different language backgrounds (Arabic, Azerbaijani, Bulgarian, Chinese, Dutch, English, Estonian, Farsi, French, Georgian, Indonesian, Italian, Japanese, Korean, Mongolian, Norwegian, Persian, Portuguese, Russian, Serbian, Serbo-Croatian, South-Korean, Spanish, Turkish, Ukrainian, Hungarian, Urdu and Uzbek). Ahead of the analysis, we had to exclude eight participants who did not manage to follow the procedures of the training study (e.g., did not participate in a session), five participants who erroneously took part in two of the three experiments, and one additional participant due to technical reasons (first training session was not stored). In our final sample, 35 participants graduated from high school, 38 from university/college, and two completed elementary school. Besides the requirement of not being a native German speaker in all Experiments, in Experiment 1, we further restricted the group to participants who should not outperform the 16th percentile in the reading speed test at the beginning of the experiment. In Experiment 2, we had no further restrictions to test if the reading speed restriction could be a potential reason for the training effect. In Experiment 3, we wanted to focus on the group of language learners most like being the training target by selecting only the participants who must not have lived in Germany for longer than 2 years. We advertised the study at the Goethe University Frankfurt via social media, e-mails, and flyers. Participants gave their written informed consent and received student credits or financial compensation (10€/h) as an incentive for participating in the experiment.

### Procedure and material

The core of the lexical categorization training is a lexical decision task, including feedback on whether the response was correct (see Fig. 2A). Participants must evaluate a visually presented letter string as a real word in a lexical decision task. We assume that executing lexical decisions trains the categorization process implemented in the left-ventral occipito temporal cortex as described by the LCM^5^. Experiment 1 was the first lexical categorization training pilot. Experiments 2 and 3 compared the lexical categorization training with other procedures in a randomized, controlled fashion. Note that we focus on the lexical categorization training to implement the diagnostic procedure; to date, the number of participants is insufficient for the control training procedures. In Experiment 2, we implemented a phonics task^25,48^. In Experiment 3, we tested the comparison with a variant of the lexical categorization training (i.e., with changing fonts) inspired by the finding that an orthographic prediction error representation is the basis of efficient visual word recognition^34^. To implement a within-participant design, in experiments 2 and 3, readers trained their lexical categorization capabilities in one of two randomly assigned training weeks. Participants trained with the control procedure the other week and had to wait at least 14 days between the 2 training weeks.

The succession within one training week was as follows: First, a reading speed assessment, then three sessions of the training task (lexical decision, phonics, or lexical decisions with changing fonts), including 1600 trials (see Fig. 2B). After the final training session, we conducted a post-training assessment to compare the reading speed before and after the training. Critical here is that for predicting the outcome (i.e., the reading speed change), we used only the pre-training reading speed data and the data from the first session of lexical decisions (i.e., dashed line frame marking in Fig. 2B). Thus, we inferred the reading speed level after completing the training procedure.

#### Pre- and post-diagnostics

We measured the reading speed with the adult version of *Salzburger Lesescreening* (SLS; unpublished adult version of ref. ^71^), a paper-and-pencil reading speed test with two available versions. Notably, the assessment measures reading on the sentence level, a more complex and naturalistic process than typically implemented in visual word recognition tasks. In this test, participants must evaluate the semantic content of as many sentences as possible within a time limit as semantically correct (e.g., “Schnee ist rot” - *Snow is red* or “In einem Wald stehen viele Bäume” - *A forest is full of trees*).

In Experiment 1, we randomized the order of both available SLS versions, i.e., one pre- and one post-training. Experiments 2 and 3 used four shorter versions of the SLS. We split the versions since we needed two more for the randomized controlled trial design. We accompanied the item reduction by reducing the time to process the test to 1 min and 30 s from 3 min of the original version. To compare the data from all three experiments, we corrected the time limit differences by duplicating the number of answers for the short version. The percentual increase in correctly processed sentences within the time limit is the outcome measure from pre- to post-training. The number of correctly answered items and errors in the SLS served as features in predicting the effect of the training procedures. In Experiment 1, we conducted the post-diagnostics on the third day immediately after the last training session. In Experiments 2 and 3, we implemented the final assessment on the fourth day, i.e., 1 day after the final training session. We accounted for this change in the design by a variable coding the design change for the predictive model.

#### Lexical categorization training

The lexical categorization training, as well as the alternative training approaches, included 1600 five-letter stimuli, of which 800 were words selected from SUBTLEX-DE^41^, 400 pseudowords (i.e., pronounceable non-words; created by changing vowels of the selected words with another vowel, for further details see ref. ^5^), and 400 consonant strings (i.e., un-pronounceable non-words; created by replacing vowels of the selected words with consonants, for further details see ref. ^5^). We selected the word stimuli by drawing them randomly from a lexicon^41^. The idea behind this procedure was that we wanted a set of stimuli representing the naturally occurring variations in word characteristics present in the lexicon implemented in our training procedure. Thus, we increased the generalizability of our features (i.e., word-likeness, represented by OLD20 based on ref. ^46^; word frequency based on ref. ^41^; the orthographic prediction error based on ref. ^34^; lexical categorization uncertainty based on the LCM from ref. ^5^). We estimated OLD20 based on all German five-letter uppercase words (*n* = 3,110; extracted from *N* = 377,524 words of the complete SUBTLEX-DE database) using the OLD20 function of the R package *vwr*^76^. As expected, the average OLD20 for pseudowords was higher than for words and lower for consonant strings.

The task was programmed in Experiment Builder software (SR-Research, Ontario, Canada), using mono-spaced Courier-New font, the first letter in uppercase (convention for German nouns), visual angle of ~0.3^∘^ per letter. We presented the letter strings in random order (different for each participant and each session) to prevent learning-based sequence effects (see Fig. 2A for the structure of the presentation). The answer was interpreted as incorrect if the participant did not respond within 10 s. Before every task session, we presented 18 stimuli to familiarize participants with the task. One session lasted about 45–60 min. We measured lexical decision response times and accuracies as performance indicators for visual word recognition.

#### Phonics and lexical categorization font training

Both control training procedures included precisely the same letter strings, except for one consonant string in the phonics training that did not include either of the used phonemes. The experimental setup was identical with minimal changes: The difference in the phonics training was that participants heard a phoneme (a, b, d, f, g, i, j, k, l, m, n, o, p, r, s, t, u, w) via headphones at the onset of the visually presented letter string. After the presentation, we asked participants to evaluate whether the phoneme was included in the written string. In half of the trials, the phonemes corresponded to a grapheme in the letter string. For the lexical categorization font training, we presented 50 blocks of 32 strings with the same font, but between blocks, we implemented a font change (i.e., each block had a different font; see Supplementary Methods 1 fonts used). Note that all procedures have been approved by the ethics committee of the psychology department at the Goethe University Frankfurt (Nr.: 2019-65).

### Analysis

We analyzed data with the R statistical software (please find all version numbers of the libraries we used in Supplementary Methods 3). We excluded response times below 300 ms and above 4000 ms. We needed to account for several issues with the SLS measurement. We had to correct one irregularity in the SLS measurement (one participant got 1:54 min instead of 1:30 min for the SLS; the score was reduced by 25%). Further irregularities need to be documented: two participants accidentally started with the second page of the SLS test in one session; one participant had a postponed SLS measurement by 3 days and one used a version twice but more than 3 weeks apart. To test the relevance of single parameters on a group-based level, we fitted the log-transformed response times with linear mixed models, considering the interindividual, inter-trial, and inter-font (specific for lexical categorization font training) variance as random effects. The core of the fixed effect structure was a three-way interaction of categorization uncertainty (LCM parameter; ref. ^5^), word-likeness (OLD20; ref. ^46^), and training across sessions. Furthermore, we included word frequency (SUBTLEX-DE; ref. ^41^), lexicality, response accuracy (“0” represents correct, “1” represents false), and whether participants conducted the procedure in the first or the second week of training due to the randomized controlled trial. We described this procedure in the preregistration of Experiment 3. Overall, we tested the change in reading speed with training by a one-sample *t*-test against zero.

#### Cross-validated diagnostic procedure

##### Cross-validation

To keep the over-fitting of our machine learning-based diagnostic procedure to a minimum, we implemented cross-validation (see Fig. 2C and Supplementary Fig. 1). Cross-validation means repeatedly splitting a dataset into a test and a training set. Then, we use the training set to fit the machine learning pipeline. The test set is left out for this process. After training the machine learning models, we can use the test set for validation as it was unseen to the trained models. In other words, we can use left-out unseen data to evaluate the prediction performance of the trained model. With this procedure, we ensured that the prediction was as unbiased as possible by the data itself (prediction models were trained and tested on two separate datasets).

Here, we implemented a leave-one-out-cross-validation procedure while using consensus-nested leave-one-out cross-validation to tune winning hyperparameters. In principle, the leave-one-out-cross-validation procedure trains all participants of a dataset except one. The one left out is then used as a test set (see Fig. 2C). We repeat this procedure to evaluate a prediction for each participant (i.e., in our case, 76x fitting of the machine learning model to predict the training outcome of one participant). This procedure allows us to rely on relatively large training sets because we only left one dataset out for the test set (each training set trained on n-1 participants). For a subset of model variations, we implemented an additional inner cross-validation loop to prevent over-fitting, as much as possible, on the level of hyperparameter tuning (e.g., which features have been selected or which machine learning method is used) aiming at finding the winning model. This inner loop is auxiliary to the leave-one-out-cross-validation (see Supplementary Fig. 1) and again implements a leave-one-out-cross-validation based on the dataset that already left out one participant (i.e., in total, the training set is N-2 and the test set is again 1). This additional loop allows us to evaluate which hyperparameters are stable across the validation loops (i.e., select the hyperparameters that have been selected most often in the inner loop; i.e., the consensus overall loops) and apply the winning variant to left-out data (see refs. ^63,64^ and the Supplemental Methods 2 for further information). Thus, with this procedure, we keep over-fitting to a minimum by relying on left-out test data for the model evaluation and, in addition, selecting hyperparameters only based on a consensus from an inner cross-validation loop (for more details, see Supplementary Methods 2 and Supplementary Fig. 1).

##### Prediction procedure

The diagnostic procedure included three levels (see Fig. 2C): feature extraction, feature selection, and fitting procedure. In the first level, we focus on feature extraction (i.e., generating informative parameters that capture individual differences well). Besides the extraction features that reflect cognitive processes underlying reading (e.g., lexical categorization, orthographic, or lexical processing), from the first training session’s response time and accuracy measures, we also included features reflecting metadata (i.e., training week) and test scores (i.e., incoming reading speed). The key to the feature extraction from reaction times was to use linear mixed models, including random slope estimates on the random effect of participants. Linear mixed models allow for explicitly fitting the hierarchical data structure of random effects. Thus, one can consider the interindividual variance of the individual slopes from fitted parameters as estimates for individual differences related to the cognitive processes associated with the included predictors (i.e., lexical categorization ability is associated with the individual slope estimate for the lexical categorization uncertainty effect; see also refs. ^5,75^; for more details, including the variation of the model structure and predictor compositions, see Supplementary Methods 2).

In the second level, we focused on selecting the relevant features (i.e., the features that lead to accurate predictions). We applied a stepwise regression procedure to filter irrelevant features (i.e., unrelated features to reduce noise and select one of several highly correlated predictors to reduce redundancy). Note that multicollinearity is not problematic for machine learning as we do not draw statistical inferences from the model weights but focus on accurately predicting an outcome variable. We chose this method as it allows feature selection of not only the generated features from the first level but also the inclusion of newly generated interaction features. Thus, in the second step, we not only reduce the number of features to those relevant but also create new features based on interactions of the included from the first-level features that will be added to the selection process (for more details, see Supplementary Methods 2).

In the third level, we used the selected features to predict the reading speed increase of the lexical categorization training based on the following model types: (i) multiple regression, (ii) support vector machine with a linear, and (iii) a radial kernel, and (iv) random forest algorithm. Notably, the models were used as regression algorithms, predicting outcomes on a continuous scale. Here, we are interested in how helpful the training is. We consider the correlation, *t* value, and the mean square error of the comparison between the predicted and observed value indicators of the model fit. Multiple regression represents the most straightforward way of fitting data linearly based on the minimization of the residuals between the dependent variable and the sum of weighted variables. Support vector machine fits a line in a multidimensional space, minimizing the distance between the fitted line and the observed data points. The linear kernel restricts the line to be linear. For the radial kernel, the line can be curved or, in other words, non-linear. The random forest algorithm is based on combining randomly drawn decision trees (for more details, see Supplementary Methods 2).

### Reporting summary

Further information on research design is available in the Nature Research Reporting Summary linked to this article.

### Supplementary information

Supplementary material

Reporting Summary

## Data Availability

The data for all studies are available at https://osf.io/3hydt/(10.17605/OSF.IO/3HYDT).

## Code availability

The analysis scripts used for all studies are available at https://osf.io/3hydt/(10.17605/OSF.IO/3HYDT).
