# Supplementary material for: Investigating lexical categorization in reading based on joint diagnostic and training approaches for language learners
Source: NPJ Sci Learn. 2024 Apr 10;9:29. doi: 10.1038/s41539-024-00237-7 (PMC11006909; doi:10.1038/s41539-024-00237-7)
Supplement: Supplementary file 1 — Supplementary material [file 41539_2024_237_MOESM1_ESM.pdf]

Supplementary Information

Supplementary Methods 1

We used the following 50 fonts: Algerian, Andalus, Arial, Arial Rounded MT Bold, Baskerville Old Face, Bauhaus 93, Berlin Sans FB, Bernard MT Condensed, Bodoni MT, Bodoni MT Poster Compressed, Bodoni Poster, Book Antiqua, Bradley Hand ITC, Britannic Bold, Broadway, Brush Script MT, Century Gothic, Chicago, Chiller, Clarendon Light, Colonna MT, Comic Sans MS, Copperplate33bc, Courier, Curlz MT, Edwardian Script ITC, Engravers MT, Eras Bold ITC, Felix Titling, Forte, French Script MT, Harlow Solid Italic, Harrington, High Tower Text, Informal Roman, Jokerman, Juice ITC, Kristen ITC, LilyUPC, Magneto, Matura MT Script Capitals, Mona Lisa Recut, OCR A Extended, Old English Text MT, Oxford, Papyrus, Poor Richard, Script MT Bold, Univers.

Supplementary Methods 2

Cross-validated diagnostic procedure

Leave-one-out-cross-validation (LOOCV). The diagnostic procedure included three levels (see Fig. 1C): feature extraction, feature selection, and fitting procedure. To reduce the chance of overfitting, we created unseen test data using a cross-validation procedure, i.e., the LOOCV (see Fig. 1C). With this procedure, we ensured that the prediction was as unbiased as possible by the data itself (prediction trained on an independent dataset). At the same time, we had relatively large training sets because we only left one dataset out for the test set (each training set trained on n-1 participants). In this proof-of-concept study introducing this diagnostic machine learning approach, we focused on optimizing the prediction method and ran multiple models with different hyperparameters.

Consensus-nested LOOCV.

To prevent overfitting on the level of hyperparameters (e.g., which features have been selected or which machine learning method is used), we implemented an additional cross-validation loop supplementary to the LOOCV (see Supplementary Fig. 1). This additional loop allows us to evaluate the hyperparameters and apply the winning variant to left-out data. To this end, we ran a nested cross-validation procedure ((1; 2)) in which the first step consisted of tuning hyperparameters by the leave-one-out method again; however, this time within nested inner loops while subsequently holding one dataset entirely out. This step follows the goal of selecting the best parameter combination for the prediction in the outer loop. The hyperparameter selection across all inner loops was based on their consensus. Thus, we can call the procedure the consensus nested cross-validation, i.e., selecting hyperparameters of the prediction model that produced the most accurate prediction (i.e., the highest correlation between predicted and observed).

Due to this method's high computational demand (i.e., 75 leave-one-out validations within each LOOCV), we restricted the number of hyperparameter combinations to 144 instead of 720 combinations (4 predictor compositions x 9 feature selection criteria x 4 fitting procedures; see Supplementary Fig. 2-4) by focusing only on one model structure. Specifically, we used only a simple mixed model structure ($lmer(log(responsetime)$ $[predictorcomposition]$ $+$ $(effect|subject), [data])$ during feature extraction as we could not observe any benefit from more complex model structures (e.g., by adding the random effect of items or adding behavioral measures; see Supplementary Figure 2). After that, we applied the most often winning model from the inner loops to predict the dataset outside the loop.

We are aware that we would optimally need an additional held-out data set that stays completely unseen during the hyperparameter tuning, and consensus nested cross-validation needs to be better established. Still, we can justify our choice by findings that nested cross-validation is largely robust against overfitting ((2)) and that similar accuracy was found for standard nested cross-validation and consensus nested cross-validation ((1)). We preferred consensus nested cross-validation because standard nested cross-validation is not perfectly applicable here as we aim at dimensional and not categorization predictions. When different models across participants are applied, the considerable differences in the feature extraction, selection, and fitting can result in inconsistent predictions and deteriorate the overall prediction strength (i.e., correlation predicted vs. observed).

Cross-validation for categorization. We explored whether the best dimensional prediction model that numerically predicted the training success (e.g., expected reading speed increase of 10 %) could also be used to categorize responders vs. non-responders (i.e., as a categorization model). A categorization criterion typically optimizes the tradeoff of sensitivity and specificity (as high sensitivity as possible – the proportion of correctly identified responders, and as high specificity as possible – the proportion of correctly identified non-responders; i.e., specificity and sensitivity optimally balanced). Here, we tested within the inner loops of nested cross-validation, which predicted training benefit should serve as a criterion. In the next step, we aggregated these values across the loops based on a median and applied the resulting criterion value (decision boundary; i.e., selected if expected training success is larger than 13.5%).

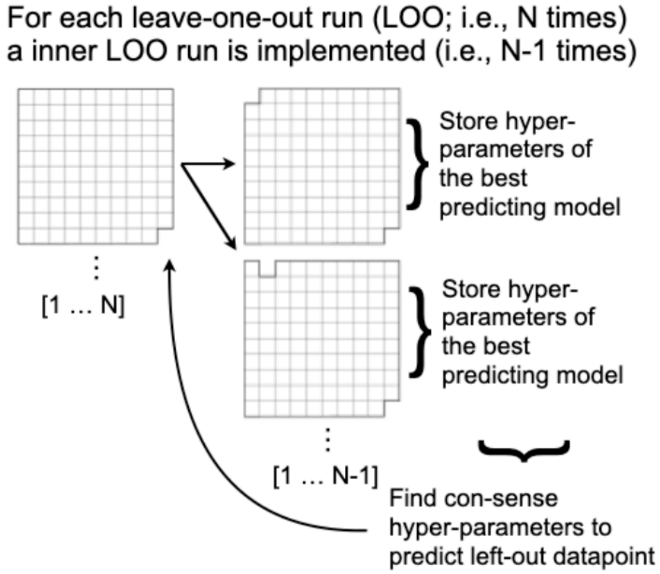

Supplementary Figure 1: Schematic depiction of the consensus nested cross-validation to tune the best hyperparameter combinations (e.g., which prediction method is best). For each leave-one-out run, we implemented an inner loop. Within these inner loops, we again ran leave-one-out procedures to find the pipeline that resulted in the highest correlation. Across all inner loops, we searched for the hyperparameter combination selected most often (i.e., find a consensus on the pipeline for the outer loop). We use the consensual selected pipeline for the predictions in the outer loop.

Prediction procedure

Level 1 – Feature extraction. The first step of our machine learning pipeline is extracting features from the first training session's response time and accuracy measures. Also, we used metadata like training week or incoming reading speed as additional features. To extract features from reaction times, we fitted linear mixed models, including specific random slope estimates on the random effect of participants. Linear mixed models allow for the explicit fitting of the hierarchical data structure of random effects. Thus, one can consider the interindividual variance and the individual slope on fitted parameters.

Exploiting this possibility, we fitted for each parameter or interaction of interest a separate model, extracting the corresponding individual slope (see Supplementary Figure 7A). For example, when we would hypothetically include parameters a, b, and c as predictors and the interaction of a and b, we would need to fit four models to get the individual estimates on these parameters. Running multiple models and not a single model that includes all random effects (that might be more intuitive) resulted in higher reliability of the results (more observation within each cluster). The interindividual variance was thereby maximal, and the shrinkage, typically implemented for complex models, is restricted (see (3)). Thus, we used the individual slope estimates for each parameter and interaction of interest as separate features.

We based the predictor composition in the linear mixed models on the assumptions from the LCM and identical to the group-based analyses described above. Notably, we included the interaction of categorization difficulty and word likeness as they are both supposed to be relevant for the pre-semantic word processing in the visual word form area (see (4)). For our basic model structure, we fitted log-transformed response times and added one random effect, taking the inter-individual hierarchical structure into account. In the context of the hyperparameter search, we ran 20 models varying the model structures and predictor compositions (5 different structures times four different compositions). With these variations, we aimed (i) to find the optimal model for outcome prediction and (ii) to ensure the robustness of our interpretations, as all models showed certain similarities (see all combinations in Supplementary Table 1).

Varying predictor composition comprised especially adding interactions to the above-described basic variant. Furthermore, we added the log-transformed sequence index in all alternative models, capturing the training effect within one session. Focusing on the interactions, we tested 3-way interactions of categorization difficulty and word-likeness with (i) lexicality, (ii) word frequency, and (iii) sequence index. The pre-lexical processing is likely affected by whether the letter string represents any semantics or not (interaction with lexicality) or whether the word can be

Supplementary Table 1
Table with combinations of calculated predictor compositions and model structures resulting in 20 different models.

Predictor composition	Model structure
1. Basic – interaction of categorization difficulty and word-likeness *categorization difficulty * old20 + word frequency + lexicality + errors + order*	1. Basic type *log(response time) ~ [predictor composition] + (effect | subject)*
2. 3-way interaction with lexicality and sequence index added	2. Random effect of items added
3. 3-way interaction with word frequency and sequence index added	3. Behavioral measures added
4. 3-way interaction with sequence index added	4. Subset for correct items
	5. Binomial distribution of errors

found very fast or very slow in the mental lexicon (interaction with word frequency). Finally, we might achieve better predictions by estimating the training effect on word-likeness and categorization difficulty within the first session (interaction with sequence index).

Varying the model structure, we added to the (i) basic structure, (ii) the random effect of items (i.e., which letter string was presented), (iii) behavioral measures from the pre-diagnostics to the response time data (SLS correct pre-diagnostics + SLS errors pre-diagnostics + design), (iv) we subset our data to correct items only and (v) fitted the binomial distribution of correctness of the responses with generalized linear mixed models (*glmer* instead of the *lmer* function). Finally, independently from extracting features from the random effects of the mixed models, we accounted for the pre-training reading level (by the number of correctly and falsely answered items in SLS), the training week (week 1 or 2), and the design variable (indicating if the post-diagnostic has been implemented on day 3 or 4).

Level 2 – Feature selection. We applied a stepwise regression procedure to filter irrelevant features and reduce noise (i.e., unrelated features) and redundancy (i.e., select one of several highly correlated predictors). The selection starts with the intercept model, and predictors are sequentially added based on the fit improvement measured by Akaike Information Criterion (AIC, (5; 6)). Our maximal model included all features and their 2-way interactions. The procedure examined all variables at each stage of the selection process to identify if a feature could be excluded without deteriorating the model fit. The search for relevant features ends when the fit cannot be improved anymore (i.e., AIC reduction when additional parameters are added). With an evaluation based on AIC, we aim for a simple model as AIC penalizes model complexity. The crucial factor why we chose this feature selection method was that it allows the interaction of features.

The stepwise regression ran multiple times (i.e., 75 times - number of training sets * 74 - number of stepwise regressions per training set; 5,550 times in total per hyperparameter combination) within the leave-one-out cross-validation method (see Supplementary Figure 7B). Subsequently, we counted how often the solution of the stepwise regression identified a feature to be relevant. If it was more often than a cutoff value across the n-1 runs (testing for cutoff values 0, 5, 10, 20, 30, 40, 50, 60, 70), the feature was selected to be used by the fitting procedures. In this way, we found a robust set of features that should likely not overfit. Note that the feature selection had to be run for all training sets of each model variant (i.e., for the outer loop: 75 training sets *74 runs of stepwise regression *20 models variants*9 cutoff values*4 fitting procedures = 3,996,000). In the inner loop, we omitted running stepwise regression 73 times within each inner training set to save computational resources (i.e., it would be 75*74*73 = 405,105 multiple regression per hyperparameter combination instead of 5,550). Thus, we use feature selection identical to the outer loop (i.e., across all 74 datasets within each training set). This simplification hardly influences the results, as only features identified as relevant just as often as the cutoff value would be added or removed differently.

Level 3 – Fitting procedure. We used the selected features to predict the reading speed increase of the lexical categorization training based on the following model types: (i) multiple regression, (ii) support vector machine with a linear and (iii) a radial kernel, and (iv) random forest algorithm. Notably, the models were used as regression algorithms, predicting outcomes on a continuous scale. We are interested in which amount the training might be helpful. We consider the correlation, t-value, and the mean square error of the comparison between the predicted and observed value indicators of the model fit. We tested all four fitting procedures for each of the 20 model variants.

Multiple regression represents the most straightforward way of fitting data linearly based on the minimization of the residuals between the dependent variable and the sum of weighted variables. Support vector machine fits a line in a multidimensional space, minimizing the distance between the fitted line and the observed data points. The linear kernel restricts the line to be linear. For the radial kernel, the line can be curved or, in other words, non-linear. Besides defining the kernel, we kept standard settings from the package e1071 ((7)). The random forest algorithm is based on combining randomly drawn decision trees. We chose to draw 500 decision trees with three features each (m = 3 in the bag; the remaining features out of the bag are used to evaluate the tree). Finally, to ensure the prediction was independent of the randomization, we ran the random forest model 30 times and averaged the predictions.

Supplementary Methods 3

Software specifications: R statistical software version 3.5.1 (R Core Team, 2018), lme4 version 1.1.21 (Bates et al., 2019), Matrix version 1.2.17 (package needed for lme4) (Bates Maechler, 2017), e1071 version 1.7.2 (Meyer et al., 2019), MASS version 7.3.51.4 (Ripley et al., 2019), randomForest version 4.6.14 (Liaw & Wiener, 2018), reshape2 version 1.4.3 (Wickham, 2017), broom version 0.5.4 (Robinson Hayes, 2020), and ggplot2 version 3.2.1 (Wickham et al., 2019).

Supplementary Table 2

Results of preregistered analysis of training in reaction times of Experiment 3 (lexical categorization training: LC vs. lexical categorization font training: LCf) and the results from the lexical categorization training including all participants.

	LCf (n=32)			LC (n=32)			LC (n=76)		
Parameter	*ES*	*SE*	*t*	*ES*	*SE*	*t*	*ES*	*SE*	*t*
Intercept	7.03	0.09	77.99	6.67	0.09	75.06	6.80	0.05	127.61
LC uncertainty (LC u.)	0.12	0.01	9.74	0.12	0.01	9.78	0.13	0.01	11.38
word-likeness	-0.03	0.01	-5.60	-0.03	0.01	-5.05	-0.03	0.01	-5.71
training session	-0.03	0.00	-9.75	-0.02	0.00	-7.87	-0.04	0.00	-20.66
lexicality	0.04	0.01	7.02	0.04	0.01	7.15	0.04	0.01	8.00
error trial	0.03	0.00	9.79	0.03	0.00	9.00	0.01	0.00	6.57
frequency	-0.07	0.00	-14.17	-0.07	0.00	-14.10	-0.08	0.00	-16.68
test week	-0.19	0.06	-3.41	0.01	0.06	0.26	-0.06	0.04	-1.63
LC u. x word-likeness	-0.16	0.01	-12.47	-0.16	0.01	-12.20	-0.18	0.01	-14.60
LC u. x session	-0.01	0.00	-2.28	-0.01	0.00	-1.58	-0.01	0.00	-2.39
word-likeness x session	0.00	0.00	0.16	0.00	0.00	0.29	0.00	0.00	0.29
LC u. x word-likeness x session	0.02	0.00	3.97	0.00	0.01	-0.61	0.00	0.01	2.23

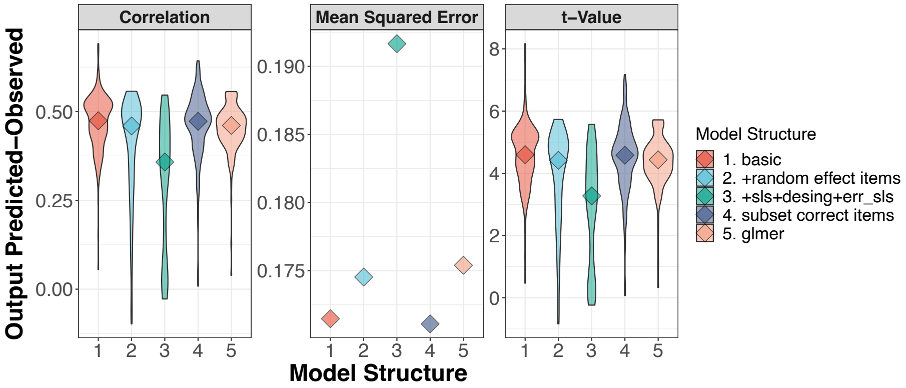

Supplementary Figure 2: Comparison of model structures in the 720 fitted models. Correlation, mean squared error, and t-value of the correlation are considered as the output variables. Note that violin plots for the mean square error are omitted for the partly immensely high values.

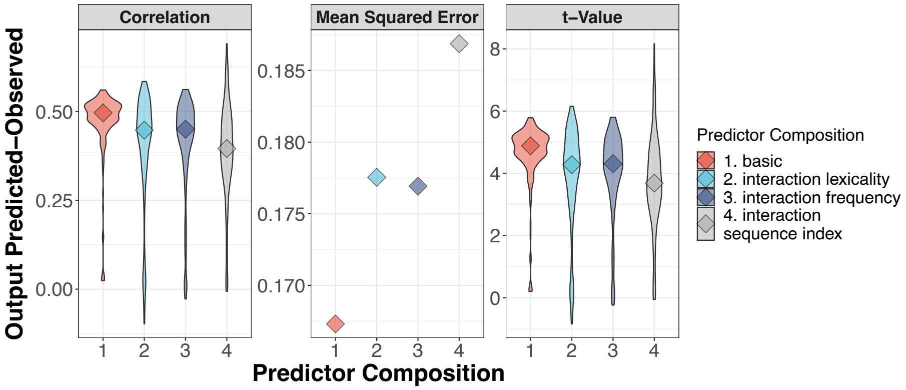

Supplementary Figure 3: Comparison of predictor composition in the 720 fitted models. Correlation, mean squared error, and t-value of the correlation are considered as the output variables. Note that violin plots for the mean square error are omitted for the partly immensely high values.

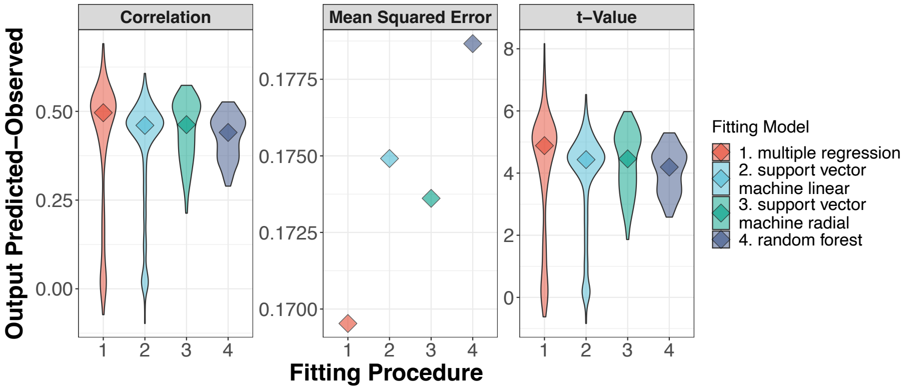

Supplementary Figure 4: Comparison of fitting procedures in the 720 fitted models. Correlation, mean squared error, and t-value of the correlation are considered as the output variables. Note that violin plots for the mean square error are omitted for the partly immensely high values.

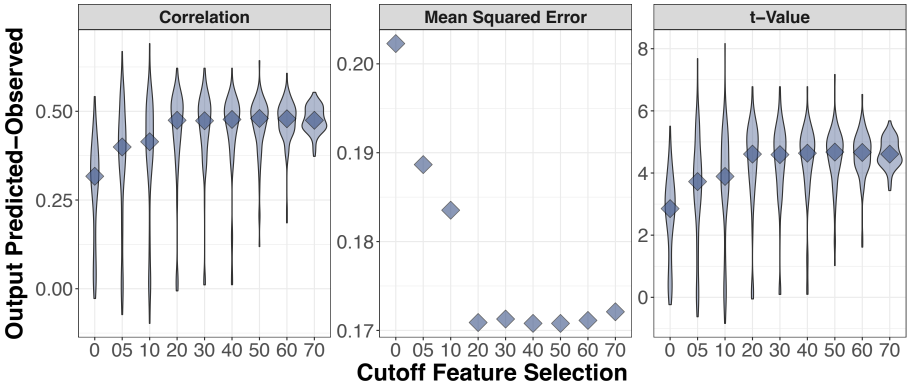

Supplementary Figure 5: Comparison of cutoff for feature selection in the 720 fitted models. Correlation, mean squared error, and t-value of the correlation are considered as the output variables. Note that violin plots for the mean square error are omitted for the partly immensely high values.

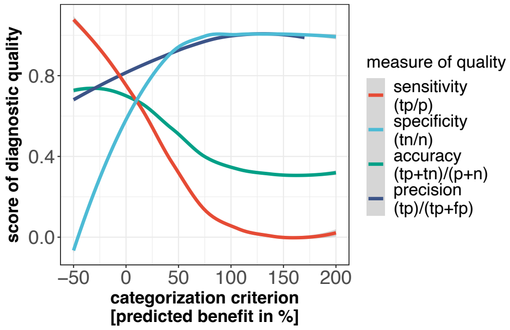

Supplementary Figure 6: Changes of sensitivity, specificity, accuracy, and precision based on the categorization criterion. tp = true positive, p = positive, tn = true negative, n = negative, fp = false positive.

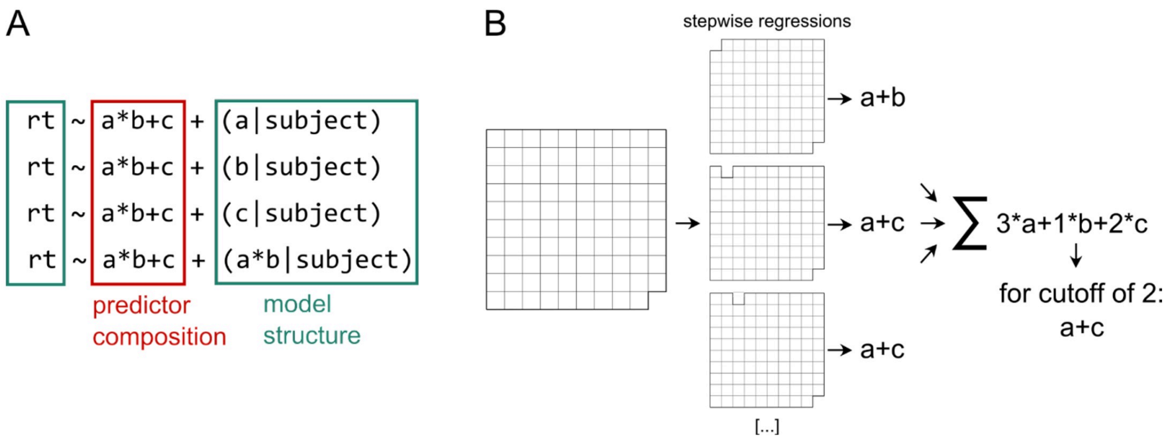

Supplementary Figure 7: Diagnostic procedure, Level 1 and 2. A) Level 1. Extracting features from random effects. All effects were fitted for an individual estimate in a separate model. Furthermore, we varied the included features (predictor composition) and the dependent variable, resp. the data and random effect structure (model structure). Combinations are listed in Table 1. B) Level 2. We used the leave-one-out method within each training data set to get a robust set of selected features. This led to 74 preselected feature sets. We counted how often each specific feature was selected and included it only when it exceeded a cutoff value in the final feature set. This procedure was simplified for the inner loop of the nested cross-validation.

Supplementary references

[1] Parvandeh, S., Yeh, H.-W., Paulus, M. P. & McKinney, B. A. Consensus features nested cross-validation. *Bioinformatics* **36**, 3093–3098 (2020). URL https://doi.org/10.1093%2Fbioinformatics%2Fbtaa046.

[2] Vabalas, A., Gowen, E., Poliakoff, E. & Casson, A. J. Machine learning algorithm validation with a limited sample size. *PLOS ONE* **14**, e0224365 (2019). URL https://doi.org/10.1371%2Fjournal.pone.0224365.

[3] Clark, M. Michael clark: Shrinkage in mixed effects models (2019). URL https://m-clark.github.io/posts/2019-05-14-shrinkage-in-mixed-models/.

[4] Gagl, B. *et al.* The lexical categorization model: A computational model of left ventral occipito-temporal cortex activation in visual word recognition. *PLOS Computational Biology* **18**, e1009995 (2022). URL https://doi.org/10.1371%2Fjournal.pcbi.1009995.

[5] Akaike, H. A new look at the statistical model identification. *IEEE Transactions on Automatic Control* **19**, 716–723 (1974).

[6] Cavanaugh, J. E. & Neath, A. A. The akaike information criterion: Background, derivation, properties, application, interpretation, and refinements. *WIREs Computational Statistics* **11** (2019). URL https://doi.org/10.1002%2Fwics.1460.

[7] Meyer, D. e1071: Misc functions of the department of statistics, probability theory group. *CRAN* (2019).
