# Supplementary material for: Investigating lexical categorization in reading based on joint diagnostic and training approaches for language learners
Source: NPJ Sci Learn. 2024 Apr 10;9:29. doi: 10.1038/s41539-024-00237-7 (PMC11006909; doi:10.1038/s41539-024-00237-7)
Supplement: Supplementary file 2 — Reporting Summary [file 41539_2024_237_MOESM2_ESM.pdf]

nature portfolio

Corresponding author(s): Benjamin Gagl

Last updated by author(s): Dec 4, 2023

Reporting Summary

Nature Portfolio wishes to improve the reproducibility of the work that we publish. This form provides structure for consistency and transparency in reporting. For further information on Nature Portfolio policies, see our Editorial Policies and the Editorial Policy Checklist.

Statistics

For all statistical analyses, confirm that the following items are present in the figure legend, table legend, main text, or Methods section.

n/a	Confirmed	
☐	☒	The exact sample size (*n*) for each experimental group/condition, given as a discrete number and unit of measurement
☐	☒	A statement on whether measurements were taken from distinct samples or whether the same sample was measured repeatedly
☐	☒	The statistical test(s) used AND whether they are one- or two-sided<br>*Only common tests should be described solely by name; describe more complex techniques in the Methods section.*
☐	☒	A description of all covariates tested
☐	☐	A description of any assumptions or corrections, such as tests of normality and adjustment for multiple comparisons
☐	☒	A full description of the statistical parameters including central tendency (e.g. means) or other basic estimates (e.g. regression coefficient) AND variation (e.g. standard deviation) or associated estimates of uncertainty (e.g. confidence intervals)
☒	☐	For null hypothesis testing, the test statistic (e.g. *F*, *t*, *r*) with confidence intervals, effect sizes, degrees of freedom and *P* value noted<br>*Give P values as exact values whenever suitable.*
☒	☐	For Bayesian analysis, information on the choice of priors and Markov chain Monte Carlo settings
☐	☒	For hierarchical and complex designs, identification of the appropriate level for tests and full reporting of outcomes
☐	☒	Estimates of effect sizes (e.g. Cohen's *d*, Pearson's *r*), indicating how they were calculated

Our web collection on statistics for biologists contains articles on many of the points above.

Software and code

Policy information about availability of computer code

Data collection	The task was programmed in Experiment Builder software (SR-Research, Ontario, Canada), that also allowed to store the collected data in a form that allowed data processing with R.
Data analysis	Data analysis was implemented in R and all scripts made available in the respective repository at https://osf.io/3hydt/

For manuscripts utilizing custom algorithms or software that are central to the research but not yet described in published literature, software must be made available to editors and reviewers. We strongly encourage code deposition in a community repository (e.g. GitHub). See the Nature Portfolio guidelines for submitting code & software for further information.

Data

Policy information about availability of data

All manuscripts must include a data availability statement. This statement should provide the following information, where applicable:

- Accession codes, unique identifiers, or web links for publicly available datasets
- A description of any restrictions on data availability
- For clinical datasets or third party data, please ensure that the statement adheres to our policy

DOI 10.17605/OSF.IO/3HYDT

Research involving human participants, their data, or biological material

Policy information about studies with human participants or human data. See also policy information about sex, gender (identity/presentation), and sexual orientation and race, ethnicity and racism.

Reporting on sex and gender	We did not collect data on sex or gender and did not include any related analysis.
Reporting on race, ethnicity, or other socially relevant groupings	We did not collect data on race, ethnicity or socio-economic status and did not include any related analysis.
Population characteristics	We collected data from a group of non-German native speakers willing to learn German as they all stayed in Germany at the time. We collected the language background and their nationalities. We documented all this information in the participant section (2.1.).
Recruitment	We advertised the study at the Goethe University Frankfurt via social media, e-mails, and flyers. Participants received student credits or financial compensation (10€/h) as an incentive for participating in the experiment.
Ethics oversight	All procedures have been approved by the ethics committee of the psychology department at the Goethe University Frankfurt (Nr.: 2019-65).

Note that full information on the approval of the study protocol must also be provided in the manuscript.

Field-specific reporting

Please select the one below that is the best fit for your research. If you are not sure, read the appropriate sections before making your selection.

☐ Life sciences ☒ Behavioural & social sciences ☐ Ecological, evolutionary & environmental sciences

For a reference copy of the document with all sections, see nature.com/documents/nr-reporting-summary-flat.pdf

Behavioural & social sciences study design

All studies must disclose on these points even when the disclosure is negative.

Study description	Three training studies have been included, of which Study 2 and 3 included a control training. The behavioral data was quantitatively analyzed using statistical and machine learning methods.
Research sample	Seventy-six adult non-native German language learners participated in the three experiments (Exp. 1: 17; Exp. 2: 27; Exp. 3: 32; 17-74 years old, M = 24.41, SD = 6.89). Note that we determined the number of participants for Experiment 3 by a power analysis described in the pre-registration (https://osf.io/t58ku) based on the estimated effects of Experiments 1 and 2 (Cohens d = 0.62; Power of 92%). Participants had no history of linguistic or neurological diseases and came from 28 different language backgrounds (Arabic, Azerbaijani, Bulgarian, Chinese, Dutch, English, Estonian, Farsi, French, Georgian, Indonesian, Italian, Japanese, Korean, Mongolian, Norwegian, Persian, Portuguese, Russian, Serbian, Serbo-Croatian, South-Korean, Spanish, Turkish, Ukrainian, Hungarian, Urdu, and Uzbek).
Sampling strategy	We determined the number of participants for Experiment 3 by a power analysis described in the pre-registration (https://osf.io/t58ku) based on the estimated effects of Experiments 1 and 2 (Cohens d = 0.62; Power of 92%).
Data collection	All three studies started and ended with a assessment including a paper-pencil reading speed measurement (SLS; see section 2.2.1). in between participants completed three sessions of a computerized training procedure.
Timing	We collected all data from 2016 to 2018.
Data exclusions	We implemented a outlier correction that removes all participants +- 2 standard deviations as defined in the preregistration of Study 3, but note we provide one analysis that includes the ourliers and one that excludes them. Showing the same results (see Table 1).
Non-participation	Ahead of the analysis, we had to exclude 8 participants who did not manage to follow the procedures of the training study (e.g., did not participate in a session), 5 participants who erroneously took part in two of the three experiments, and one additional participant due to technical reasons (first training session was not stored).
Randomization	In study 2 and 3 we randomized, for each participant, the succession of the lexical categorization training and the control procedures in a randomized controlled fashion.

Reporting for specific materials, systems and methods

We require information from authors about some types of materials, experimental systems and methods used in many studies. Here, indicate whether each material, system or method listed is relevant to your study. If you are not sure if a list item applies to your research, read the appropriate section before selecting a response.

Materials & experimental systems

n/a	Involved in the study
☒	☐ Antibodies
☒	☐ Eukaryotic cell lines
☒	☐ Palaeontology and archaeology
☒	☐ Animals and other organisms
☒	☐ Clinical data
☒	☐ Dual use research of concern
☒	☐ Plants

Methods

n/a	Involved in the study
☒	☐ ChIP-seq
☒	☐ Flow cytometry
☒	☐ MRI-based neuroimaging

Plants

Seed stocks	*Report on the source of all seed stocks or other plant material used. If applicable, state the seed stock centre and catalogue number. If plant specimens were collected from the field, describe the collection location, date and sampling procedures.*
Novel plant genotypes	*Describe the methods by which all novel plant genotypes were produced. This includes those generated by transgenic approaches, gene editing, chemical/radiation-based mutagenesis and hybridization. For transgenic lines, describe the transformation method, the number of independent lines analyzed and the generation upon which experiments were performed. For gene-edited lines, describe the editor used, the endogenous sequence targeted for editing, the targeting guide RNA sequence (if applicable) and how the editor was applied.*
Authentication	*Describe any authentication procedures for each seed stock used or novel genotype generated. Describe any experiments used to assess the effect of a mutation and, where applicable, how potential secondary effects (e.g. second site T-DNA insertions, mosiacism, off-target gene editing) were examined.*
